# A comparison of normobaric and hypobaric hypoxia effects on cerebrovascular response pre and post maximal exercise

**DOI:** 10.1113/EP093088

**Published:** 2026-01-29

**Authors:** Rachel Turner, Giovanni Vinetti, Giacomo Strapazzon, Hannes Gatterer

**Affiliations:** ^1^ Institut für Sportwissenschaft Universität Innsbruck Innsbruck Austria; ^2^ Institute of Mountain Emergency Medicine Eurac Research Bolzano Italy; ^3^ Mountain Clinic, Institute of Mountain Emergency Medicine Eurac Research Bolzano Italy; ^4^ Department of Medicine – DIMED University of Padova Padova Italy

**Keywords:** barometric pressure, cerebrovascular regulation, maximal exercise

## Abstract

A lack of consensus remains on whether normobaric hypoxia (NH) and hypobaric hypoxia (HH) may differentially impact physiological factors affecting cerebrovascular regulation, particularly with an additional strenuous exercise component. We sought to compare the acute effects of NH and HH on global cerebral blood flow (gCBF) at an altitude corresponding to 4000 m. In this randomised, single‐blind crossover study, eight lowlanders (3 females) completed three identical trials inside a hypobaric chamber: the first in normobaric normoxia, for familiarisation, followed in random order by one in NH and one in HH. In each trial, gCBF was measured at two time points via duplex ultrasound, first after 25 min of rest, and second, directly after a graded exercise test (GXT) to volitional exhaustion. Cardiorespiratory responses and cerebral oxygenation ($rS_{cO_{2}}$) were assessed during all gCBF measurements. At rest, gCBF was higher in HH than in NH (944 ± 230 vs. 883 ± 226 mL min^−1^; *P* = 0.027, respectively), whereas $rS_{cO_{2}}$ remained unchanged. Cardiorespiratory parameters did not differ, except for a reduction in the ratio of dead space to tidal volume in HH compared to NH (*P* = 0.028). Post‐GXT, no differential response between the two hypoxic conditions was found. In comparison to NH, at rest gCBF is increased in HH for a given partial pressure of inspired oxygen, a response that is subsequently abolished post maximal cycling exercise. Although subtle, this response indicates that cerebrovascular regulation is affected differently in NH and HH, despite negligible changes in ventilation, and thus, alternative explanations are explored for future investigation.

## Abstract (highlights)

- **What is the central question of this study?**
  Does hypobaria independently affect cerebrovascular regulation under hypoxic conditions, and how does this response differ with the addition of a strenuous exercise component?
- **What is the main finding and its importance?**
  Hypoxic cerebrovascular tone is subtly but distinctly affected by barometric pressure, despite negligible changes in concurrent cardiorespiratory parameters. These findings highlight the need for further mechanistic studies to determine how hypobaria may differentially impact cerebrovascular control and the subsequent relevance for both high altitude pathophysiology and therapeutic research purposes.

## 1 INTRODUCTION

Putative disparities in the physiological response to hypoxia under normobaric (NH) and hypobaric (HH) conditions remain heavily debated (Aebi et al., 2020; Coppel et al., 2015; Debevec et al., 2020; Girard et al., 2012; Loeppky et al., 1997; Richalet, 2020b). Specifically, the hypothesis that in HH the concurrent reduction in barometric pressure (*P*_B_) may present a more potent stimulus for any given reduction in inspired oxygen pressure ($P_{IO_{2}}$) continues to fuel discourse relating to an independent physiological effect of hypobaria on physiological factors that may impact cerebrovascular regulation (Aebi et al., 2020; Millet et al., 2012a; Savourey et al., 2003; Shaw et al., 2021).

In comparison to NH, HH has been purported to introduce physical gas differences, that is, reduced gas density (Loeppky et al., 1997; Ogawa et al., 2019; Tucker et al., 1983), gas diffusivity (Paganelli et al., 1975; Tucker et al., 1983), reduced air flow resistance (Angus et al., 2023; Ogawa et al., 2019) and initial differences in alveolar N_2_ kinetics (Conkin & Wessel, 2008), which may impact alveolar concentrations of O_2_ and CO_2_ and therefore ventilation and resultant blood gases. Previous work characterizing a differential breathing pattern in HH in comparison to NH at rest, that is, a higher respiratory frequency (*f*_R_), coupled with a lower tidal volume (*V*_T_), resulting in a lower overall minute ventilation (${\dot{V}}_{E}$) (Millet et al., 2012b; Savourey et al., 2003; Tucker et al., 1983), but paradoxically a greater hypoxaemia (Faiss et al., 2013; Savourey et al., 2007) and hypocapnia (Aebi et al., 2020; Faiss et al., 2013; Savourey et al., 2003). Whereas, our previous findings (Vinetti et al., 2024) are more in line with earlier studies which demonstrated no change in resting ${\dot{V}}_{E}$ (Faulhaber et al., 2020; Loeppky et al., 1996; Savourey et al., 2007), or end‐tidal carbon dioxide partial pressure ($P_{\mathrm{ETC}O_{2}}$) (Savourey et al., 2007) between conditions given a similar duration of exposure (approx. 20–60 min), thus highlighting the controversy that currently exists regarding the interchangeability of these two hypoxic conditions for either training or therapeutic purposes.

Conversely, compared to NH, HH has been associated with increased oxidative stress markers (Debevec et al., 2015; Faiss et al., 2013; Ribon et al., 2016), impaired NO bioavailability (Faiss et al., 2013) and perhaps even increased cerebrovascular reactivity to a given $P_{\mathrm{aC}O_{2}}$ (Aebi et al., 2020), all factors that could be considered to reduce vascular dilatory reserve, impact cerebrovascular regulation and may be altered with the addition of a strenuous exercise component. However, overall results remain inconsistent, making it difficult to determine whether the physiological differences observed to date are robust enough to infer a specific effect of barometric pressure at a given $P_{O_{2}}$ or are simply the result of imprecisely matched inspired oxygen tensions between conditions (Richalet, 2020a). Recurrent points of contention within the existing literature are poor standardisation between studies (Conkin & Wessel, 2008; Coppel et al., 2015; Treml et al., 2020), a lack of reproducibility (Richard et al., 2014; Savourey et al., 2007), acclimatisation bias (Coppel et al., 2015), and a relative scarcity of a directly comparative exercise component (Saugy et al., 2016).

As a result, in the present study, we aimed to evaluate whether ventilatory and cerebrovascular responses to acute hypoxia are differentially affected by normobaric or hypobaric conditions both pre and post a maximal exercise test under highly controlled conditions. Between conditions the inspiratory pressure of O_2_ ($P_{IO_{2}}$) simulating 4000 m was carefully matched. Under resting conditions, we hypothesized that in comparison to NH, HH exposure would result in a negligible change in ventilatory response, resulting in equivocal changes in global cerebral blood flow (gCBF) between these two hypoxic conditions, whereas we hypothesized that after a maximal exercise bout, during which we previously reported a higher ventilation and power output in HH (Vinetti et al., 2024), $P_{\mathrm{aC}O_{2}}$ and thus gCBF would be reduced further in HH compared to NH.

## 2 METHODS

### 2.1 Ethical approval

This study was approved by the Ethics Committee for Clinical Trials of the Autonomous Province of Bolzano, Italy (No. 92–2020) and conducted in adherence with the current version of the *Declaration of Helsinki* (except registration in a database). The data were collected as part of a larger project investigating the cardiorespiratory responses to exercise in hypobaric versus normobaric hypoxia (Vinetti et al., 2024), but the hypotheses and data are unique and not reported elsewhere. Informed consent was obtained from all participants in writing prior to study starting.

### 2.2 Subject recruitment and screening

Eight healthy lowlanders (5 males, 32 ± 5 years, 73 ± 6 kg, 177 ± 4 cm; and 3 females, 30 ± 6 years, 61 ± 8 kg, 174 ± 7 cm) completed the study in its entirety. All performed a medical screening prior to the study starting (e.g., anamnesis, resting blood pressure, resting and exercise ECG). All participants were non‐smokers, physically active (>2 training sessions/week), resident at low altitude (median 269 m, range 228–1220), with no history of systemic disease or serious high‐altitude illness. Other exclusion criteria were high altitude ancestry and exposure to >2500 m during the 4 weeks preceding the study. To ensure comparable results, subjects were also instructed to abstain from heavy exercise and to match nutrition and fluid intake in the 48 h preceding each testing session (including a light meal 2 h before).

### 2.3 Study design

Subjects completed three identical trials inside a hypobaric chamber (terraXcube, Eurac Research, Bolzano, Italy, located 250 m above sea level), the first was a familiarisation session in normobaric normoxia (NN; $P_{IO_{2}}$: 147.0 ± 0.8 mmHg), followed by one in NH ($P_{IO_{2}}$: 89.7 ± 2.0 mmHg) and one in HH ($P_{IO_{2}}$: 89.5 ± 2.3 mmHg), each separated by 2 weeks. The NH and HH trials were conducted in a randomised order, with the subjects blinded to the allocated sequence and the experimental conditions. In each trial, gCBF was measured at two time points (TP) by duplex ultrasound in a supine position, first after 25 min of supine rest at the desired $P_{IO_{2}}$ (TP1) (post a 20 supine ‘ascent’), and second, after a 3 min active cycling recovery (35 W), directly post the cessation of a graded exercise test (GXT) to volitional exhaustion (TP2). Peripheral and cerebral oxygenation, ventilatory and cardiovascular parameters were captured alongside each gCBF measurement. Overall, a 5 min average was recorded, except for the mean arterial pressure measurements (3 min average), both starting from the same time point. Figure 1 depicts the experimental design. The total time of exposure was (from start of the 25 min rest period at ‘target altitude’ to end of TP2 measurement) 106 ± 3 and 106 ± 4 min (mean ± SD) in NH and HH, respectively.

**FIGURE 1:**
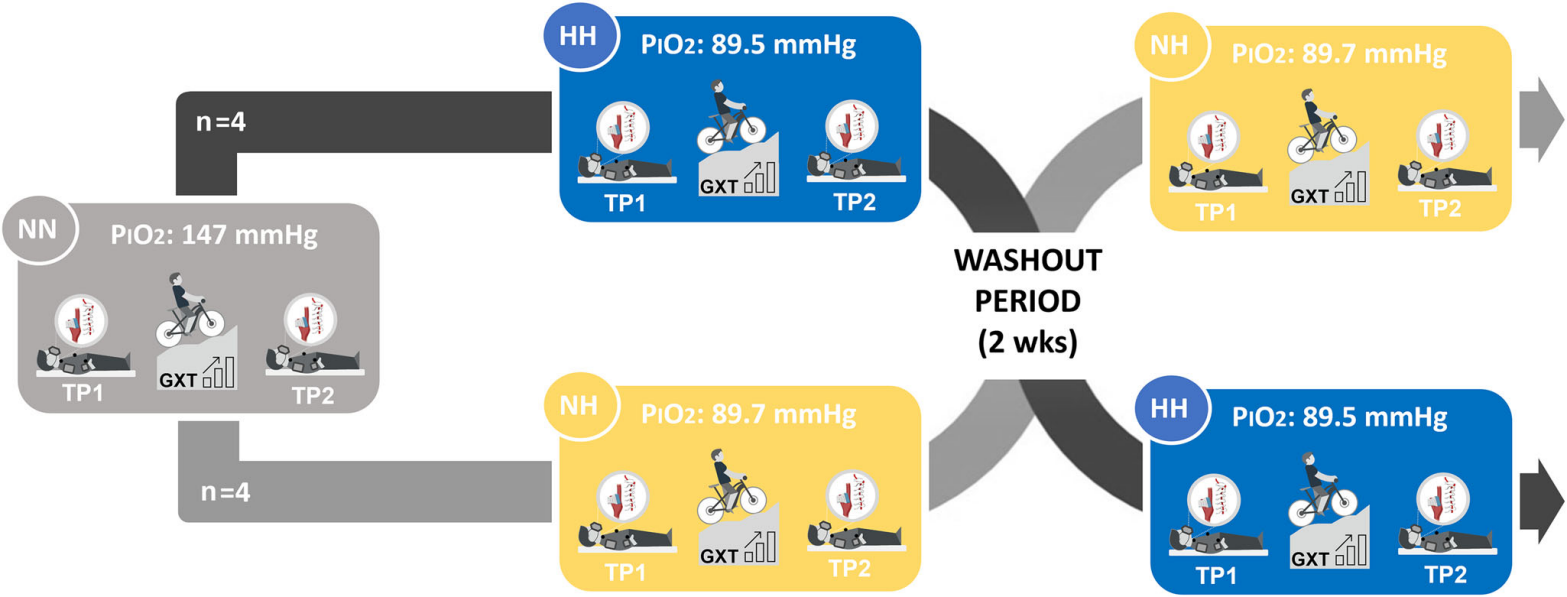
Experimental design and protocol. Within the crossover design, subjects were randomly assigned to two different hypoxic conditions at an identical $P_{IO_{2}}$ equivalent to 4000 m. All subjects started with a familiarisation session in normobaric normoxia (NN), before being randomly assigned within a matched pairs, crossover design to either normobaric hypoxia (NH: yellow) or hypobaric hypoxia (HH: blue) for the first test session. gCBF was measured at two time points by duplex ultrasound, first after 25 min of rest (TP1), and second, directly after a graded exercise test (GXT) to volitional exhaustion (TP2). Subjects then completed a 2‐week washout period before returning to repeat the other condition. All participants were blinded to hypoxic condition, including the use of a placebo decompression (cycles of decompression and re‐compression of ∼50 hPa) in the NH condition to mimic the HH ‘ascent’.

### 2.4 Measurements and procedures

#### 2.4.1 Cerebral oxygenation and blood flow

Vertebral artery (VA) and internal carotid artery (ICA) blood flows were measured in a supine position at sea level, after 25 min of NH and HH exposure at rest and after a maximal cycling exercise test to exhaustion (previously described in detail; (Vinetti et al., 2024). Measurements after the GXT were obtained after participants had completed a 3 min active recovery phase on the bicycle at 35 W and a 5 min rest phase in the supine position. Care was taken to ensure that data were being sampled at the same time point for each participant. gCBF was measured by continuous, high‐resolution sonography (CX50 POC Compact Xtreme system; Philips Healthcare, Bothell, WA, USA) with a 12 MHz broadband linear array transducer (L12‐3; Philips Healthcare, Bothell, WA, USA) on the right‐hand side. ICA imaging occurred ≥1.5 cm distal to the common carotid bifurcation and VA imaging between cervical vertebrae 4 and 5, 5 and 6, or proximal to the entry into the vertebral column. The exact location was chosen on an individual basis in an attempt to select the most reproducible measures, with the same location repeated within subjects and between sojourns. B‐mode measurements were noted to ensure consistent probe placement and depth, power, gain and dynamic range were maintained for each subject. All ultrasound recordings were screen‐captured, anonymised and stored for offline analysis. To improve the accuracy of blood flow measurements and minimise the trade‐off between B‐mode and pulsed‐wave Doppler mode, we took ICA and VA diameter and velocity measurements in quick succession (Friend et al., 2021). First, the diameter was recorded as a B‐mode image over 30 s and manually measured offline in triplicate during end‐diastole in Qlab (QLab 11.0; Philips Healthcare, Andover, MA, USA) as previously specified (Thomas et al., 2015). The velocity of red blood cells was measured from the onboard integrated, intensity weighted calculation of the time averaged mean velocity (TAMV) over 30 s at an insonation angle of 60°, with the cursor set in the centre of the vessel and the Doppler gate adjusted to the size of the vessel. Volumetric blood flow in the ICA and VA was calculated (in mL min^−1^) as ${\dot{Q}}_{\mathrm{VA}}$ or ${\dot{Q}}_{\mathrm{ICA}}$ = TAMV × [π × (diameter/2)^2^] × 60, with TAMV expressed in cm s^−1^ and artery diameters in cm (Evans, 1985). To account for MAP in our analysis of the cerebrovascular responses, volumetric vascular conductance (CVC) was calculated as ICA CVC = ${\dot{Q}}_{\mathrm{ICA}}$/MAP and VA CVC = ${\dot{Q}}_{\mathrm{VA}}$/MAP (mL min^−1^ mmHg^−1^). Global cerebral blood flow was estimated as gCBF = (${\dot{Q}}_{\mathrm{VA}}$ + ${\dot{Q}}_{\mathrm{ICA}}$) × 2 (mL min^−1^). In addition, regional cerebral oxygen saturation ($rS_{cO_{2}}$) was continuously monitored using multiwavelength near‐infrared spectroscopy (NIRS, O3 Regional Oximetry, Masimo Corporation, Irvine, CA, USA). A single adhesive sensor (adult, 40 mm source–detector spacing) was placed at a standardised fronto‐temporal position high on the forehead to minimise potential signal contamination from the frontal or sagittal sinuses.

#### 2.4.2 Cardio‐respiratory measurements

During cerebral blood flow measurements, subjects wore a face mask (Hans Rudolph 7450 V2 mask; Hans Rudolph, Shawnee, KS, USA) that was connected to a portable gas analyser (METAMAX® 3B; CORTEX Biophysik, Leipzig, Germany). Oxygen uptake, carbon dioxide output (${\dot{V}}_{O_{2}}$ and ${\dot{V}}_{CO_{2}}$), at standard temperature and pressure, dry air, stpd), respiratory exchange ratio (RER), minute ventilation (${\dot{V}}_{E}$ at body temperature and *P*_B_, saturated with water vapour, btps), tidal volume (*V*_T_), breathing frequency (*f*_R_), and end‐tidal gas partial pressures ($P_{\mathrm{ET}O_{2}}$ and $P_{\mathrm{ETC}O_{2}}$) were recorded. The ratio of dead space to tidal volume (*V*_D_/*V*_T_) was estimated using Bohr's equation, with end‐tidal CO_2_ as a surrogate of alveolar CO_2_ (Anthonisen & Fleetham, 1987). Heart rate (HR) was measured using a Bluetooth‐enabled chest stap (Polar H7 heart rate sensor; Polar Electro Oy, Kempele, Finland). Arterial oxyhaemoglobin saturation ($S_{pO_{2}}$, %) was continuously assessed non‐invasively by pulse oximetry (WristOx2 Model 3150 with 8000SM sensor; Nonin Medical, Plymouth, MN, USA). Continuous arterial blood pressure was measured separately non‐invasively by finger plethysmography (Finapres® NOVA; FMS, Enschede, The Netherlands) during TP1 measurements only. Blood pressure waveforms were sampled at 1 kHz (PowerLab 16/35 and LabChart software; ADInstriments, Dunedin, New Zealand). Mean arterial pressure (MAP) and stroke volume (SV), were derived from the arterial blood pressure waveform using the modelflow (MF) method (Wesseling et al., 1993). Cardiac output (CO) was calculated mathematically as the product of HR and SV.

#### 2.4.3 Conditions comparison

To evaluate differences between NH and HH conditions, iso‐$P_{IO_{2}}$ between NH versus HH was ensured by adjusting *P*_B_ in the hypobaric chamber or $F_{IO_{2}}$ based on the known equation ($P_{IO_{2}}$ = (*P*_B_ − 47) × $F_{IO_{2}}$), when 47 mmHg corresponds to water vapour pressure at 37°C (Conkin, 2016). Dependent on the order of exposure (i.e., NH vs. HH first), iso‐$P_{IO_{2}}$ was ensured using two different methods as previously described in detail (Vinetti et al., 2024). In brief, in the first hypoxic session, *P*_B_ (if HH) or $F_{IO_{2}}$ (if NH) was fixed for all participants to 462.3 mmHg and 13.0%, respectively, while in the subsequent crossover session, *P*_B_ or $F_{IO_{2}}$ was regulated to match the $P_{IO_{2}}$ of the first session. Overall, HH consisted of *P*_B_ 474 ± 11 mmHg and NH of $F_{IO_{2}}$ 12.9 ± 0.3% (*P*_B_ 741 ± 3 mmHg). Temperature (21.0 ± 0.1°C) and humidity (40 ± 3%) were also carefully maintained across all conditions.

#### 2.4.4 Statistics

Sample size was calculated (G*Power, V.3.1.9.4) to detect a 2% mean difference in $S_{pO_{2}}$ (SD 2%) between hypoxic conditions, as reported by Vinetti et al. (2024), resulting in *n* = 8 matched pairs (type I error of 0.05 and a statistical power of 0.8). It was assumed that from such differences in $S_{pO_{2}}$, differential respiratory and cerebrovascular responses could be directly inferred.

Statistical analysis was performed with the SPSS Statistics version 25 statistical package (IBM Corp., Armonk, NY, USA). The Shapiro–Wilk test and inspection of the data were undertaken to assess normality of all continuous data. Since we were not able to capture one ICA TAMV measurement (TP1) due to a technical fault, we used generalized estimating equations (including condition, i.e., NH and HH and test order) to evaluate differences in brain blood flow variables between hypoxic conditions and measurement time points. Differences between NH and HH for the remaining parameters were evaluated using Student's paired *t*‐test. Effect size was calculated with Cohen's *d* and classified as: 0.2–0.4 small, 0.5–0.7 medium and ≥0.8 large (Cohen, 2013). Data are presented as means ± standard deviation (SD) throughout and results are presented alongside Wald confidence intervals (CI). A two‐tailed *P*‐value <0.05 was considered statistically significant for all comparisons.

## 3 RESULTS

A total of eight subjects (three females) enrolled in the study (31 ± 5 years, 68.9 ± 8.7 kg, 1.76 ± 5.5 m). All subjects completed the NN familiarisation trial before the two hypoxic comparison trials (NH vs. HH). Regarding subject blinding, when questioned, 6/8 participants were unable to determine which hypoxic condition they were in on either visit.

Table 1 compares resting cardiorespiratory, cerebrovascular indices and $rS_{cO_{2}}$ between the NH and HH conditions (TP1), in line with NN measurements taken during the familiarisation trial. gCBF was 6.9% higher in HH than in NH at rest (944 ± 230 vs. 883 ± 226 mL min^−1^; *P* = 0.027, respectively), due to a 6.9% increase in ${\dot{Q}}_{\mathrm{ICA}}$ as a result of marked ICA vasodilation (4.0% increase in diameter) and a 7.91% increase in vascular conductance (TP1: Figure 2 and Table 1; all *P* ≤ 0.021), whereas $rS_{cO_{2}}$ remained unchanged in HH, compared to NH.

**TABLE 1:** Parameters at rest in normoxia, normobaric and hypobaric hypoxia; TP1.

	NN [95% CI]	NH [95% CI]	HH [95% CI]	*P* (NH vs. HH)	Effect size
$S_{pO_{2}}$ (%)	96.0 ± 3.6 [93.5, 98.5]	77.4 ± 3.5 [75.0, 79.8]	78.8 ± 3.8 [76.1, 81.4]	0.247	0.38
HR (min^−1^)	55.7 ± 6.6 [51.2, 60.3]	59.3 ± 7.6 [54.1, 64.6]	59.0 ± 6.1 [54.8, 63.3]	0.870	0.04
MAP (mmHg)	—	94.5 ± 11.8 [85.7, 103.3]	92.6 ± 10.2 [85.5, 99.7]	0.199	0.17
CO (L min^−1^)	—	4.5 ± 1.0 [3.8, 5.2]	4.1 ± 1.5 [3.1, 5.2]	0.575	0.29
$P_{\mathrm{ET}O_{2}}$ (mmHg)	107.5 ± 2.4 [105.8, 109.1]	51.6 ± 2.1 [50.2, 53.1]	51.7 ± 2.8 [49.8, 53.6]	0.972	0.01
$P_{\mathrm{ETC}O_{2}}$ (mmHg)	34.0 ± 1.7 [32.8, 35.1]	32.2 ± 1.6 [33.1, 33.3]	31.9 ± 1.9 [33.2, 30.6]	0.529	0.19
RER	0.85 ± 0.03 [0.83, 0.88]	0.83 ± 0.03 [0.81, 0.85]	0.82 ± 0.02 [0.81, 0.84]	0.580	0.16
${\dot{V}}_{E}$ (L min^−1^ btps)	9.4 ± 1.5 [8.3, 10.4]	9.4 ± 2.5 [7.7, 11.2]	8.8 ± 2.0 [7.5, 10.2]	0.213	0.27
*V*_T_ (L btps)	0.58 ± 0.07 [0.54, 0.63]	0.65 ± 0.1 [0.55, 0.74]	0.61 ± 0.1 [0.52, 0.70]	0.430	0.26
*f*_R_ (min^−1^)	16.2 ± 2.6 [14.4, 17.9]	15.0 ± 4.3 [12.0, 18.0]	15.2 ± 5.3 [11.5, 18.8]	0.896	0.03
${\dot{V}}_{E}/{\dot{V}}_{O_{2}}$	32.2 ± 3.0 [30.1, 34.3]	32.2 ± 2.6 [30.4, 34.0]	30.9 ± 1.6 [29.8, 32.1]	0.213	0.58
${\dot{V}}_{E}/{\dot{V}}_{CO_{2}}$	37.7 ± 3.0 [35.6, 39.8]	38.9 ± 2.6 [37.1, 40.7]	37.6 ± 1.7 [36.4, 38.8]	0.275	0.60
${\dot{V}}_{A}$ (L min^−1^ btps)	6.3 ± 1.0 [5.6, 7.0]	6.5 ± 1.7 [5.3, 7.7]	6.3 ± 1.2 [5.5, 7.1]	0.640	0.12
*V*_D_/*V*_T_ (%)	32.3 ± 3.6 [34.8, 29.8]	30.8 ± 5.0 [27.4, 34.3]	27.8 ± 4.6 [24.5, 31.0]	**0.028**	0.64
gCBF (mL min^−1^)	807 ± 181 [681, 933]	883 ± 226 [727, 1040]	944 ± 230 [773, 1115]	**0.027**	0.27
${\dot{Q}}_{\mathrm{VA}}$ (mL min^−1^)	130 ± 35 [105, 155]	153 ± 44 [122, 183]	157 ± 46 [124, 189]	0.573	0.09
${\dot{Q}}_{\mathrm{ICA}}$ (mL min^−1^)	273 ± 66 [227, 319]	289 ± 78 [250, 342]	309 ± 75 [274, 356]	**< 0.001**	0.26
VA TAMV (cm s^−1^)	18.8 ± 3.4 [16.4, 21.2]	19.5 ± 3.5 [17.1, 21.8]	19.5 ± 3.6 [16.9, 22.0]	0.988	0.00
ICA TAMV (cm s^−1^) (*n* = 7)	28.0 ± 6.6 [23.4, 32.6]	29.0 ± 6.7 [24.2, 33.7]	28.4 ± 5.9 [24.0, 32.7]	0.429	0.09
VA diameter (cm)	0.38 ± 0.03 [0.36, 0.40]	0.41 ± 0.04 [0.38, 0.43]	0.41 ± 0.03 [0.39, 0.43]	0.641	0.10
ICA diameter (cm)	0.46 ± 0.05 [0.42, 0.49]	0.46 ± 0.05 [0.43, 0.50]	0.48 ± 0.03 [0.46, 0.50]	**0.021**	0.42
VA_CVC_ (mL min mmHg)	—	1.65 ± 0.49 [1.29, 2.01]	1.65 ± 0.49 [1.31, 1.98]	0.670	0.10
ICA_CVC_ (mL min mmHg)	—	3.06 ± 1.00 [2.32, 3.81]	3.31 ± 0.99 [2.57, 4.04]	**0.016**	0.24
$rS_{cO_{2}}$ (%)	—	55.6 ± 3.5 [53.2, 58.0]	56.8 ± 2.0 [55.4, 58.2]	0.102	0.43

Data are presented as means ± standard deviation; *n* = 8 except for HH ICA TAMV, ${\dot{Q}}_{\mathrm{ICA}}$ and gCBF where *n* = 7. *P*‐values result from comparisons between NH and HH and *P* < 0.05 was considered significant and marked in bold. ES, effect size (Cohen's *d*); CI, confidence intervals (Wald). Abbreviations: CO, cardiac output; CVC, cerebrovascular conductance; *f*_R,_ respiratory frequency; gCBF, global cerebral blood flow; HH, hypobaric hypoxia; HR, heart rate; ICA, internal carotid artery; MAP, mean arterial pressure; NH, normobaric hypoxia; NN, normobaric normoxia; $P_{\mathrm{ETC}O_{2}}$, end‐tidal carbon dioxide partial pressure; $P_{\mathrm{ET}O_{2}}$, end‐tidal oxygen partial pressure; ${\dot{Q}}_{\mathrm{ICA}}$, ICA blood flow; ${\dot{Q}}_{\mathrm{VA}}$, VA blood flow; RER, respiratory exchange ratio; $rS_{cO_{2}}$, regional cerebral oxygen saturation; $S_{pO_{2}}$, peripheral oxygen saturation; TAMV, time average mean velocity; TP1, time point 1 (rest); VA, vertebral artery; ${\dot{V}}_{A}$, alveolar ventilation; ${\dot{V}}_{CO_{2}}$, carbon dioxide output; *V*_D_, ventilatory dead space; *V*_D_/*V*_T_, ratio of dead‐space to tidal volume; ${\dot{V}}_{E}$, minute ventilation; ${\dot{V}}_{O_{2}}$, oxygen uptake; *V*_T_, tidal volume.

**FIGURE 2:**
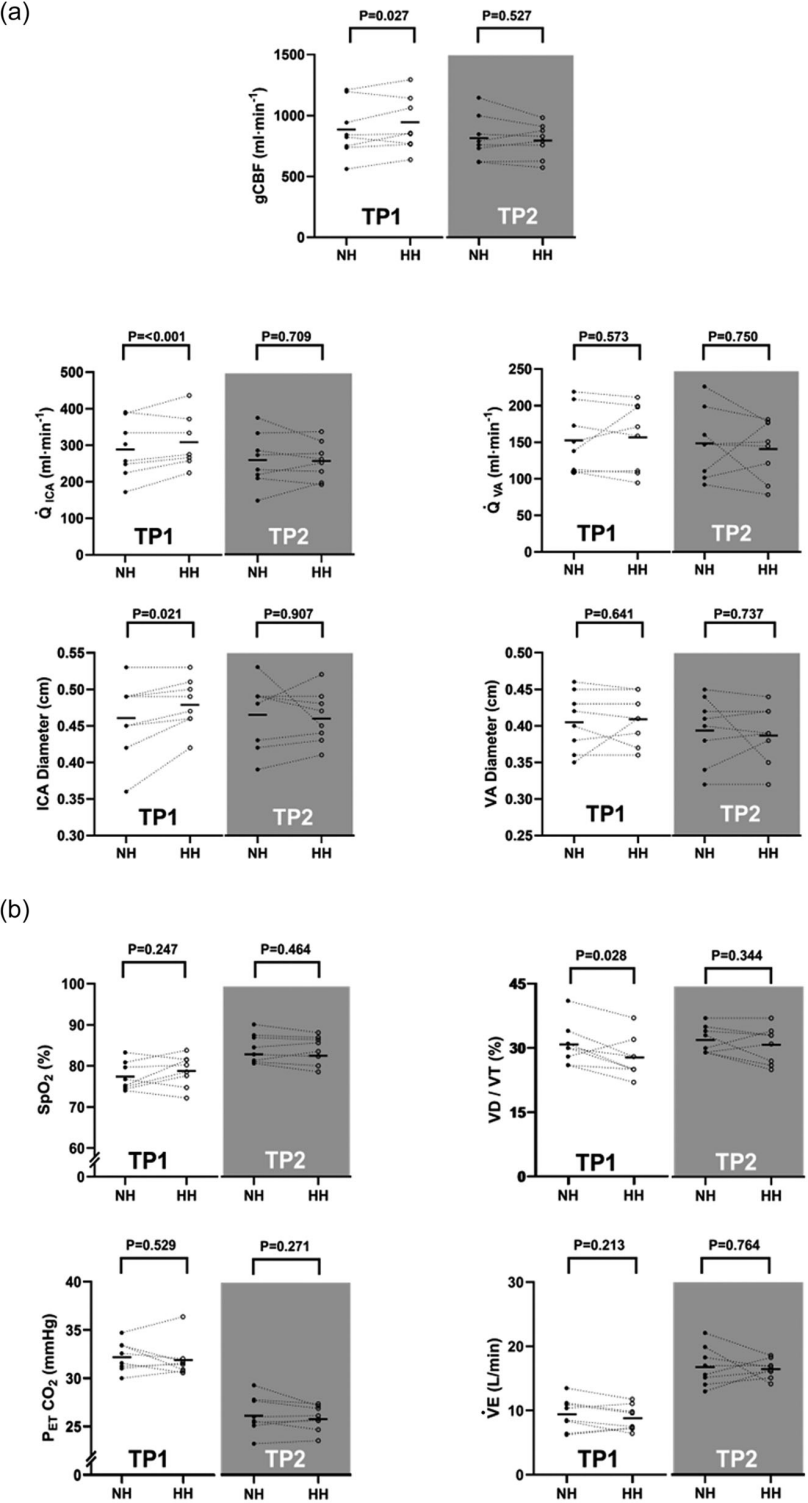
Comparison of cerebrovascular measures (a) and cardiorespiratory measures (b) in hypobaric hypoxia (HH) and normobaric hypoxia (NH) both pre (TP1) and post (TP2) a graded exercise test. *n* = 8, except for HH TP1 ${\dot{Q}}_{\mathrm{ICA}}$ and gCBF measurement where *n* = 7. Small circular symbols represent individual data points, with dotted lines tracking within‐subject changes. Bars represent the mean at each time point. *P*‐values refer to hypoxic condition comparisons at TP1 and TP2 and *P* < 0.05 is considered significant. gCBF, global cerebral blood flow; ICA, internal carotid artery; $P_{\mathrm{ETC}O_{2}}$, end‐tidal carbon dioxide partial pressure; ${\dot{Q}}_{\mathrm{ICA}}$, internal carotid artery blood flow; ${\dot{Q}}_{\mathrm{VA}}$, vertebral artery blood flow; $S_{pO_{2}}$, peripheral oxygen saturation; VA, vertebral artery; *V*_D_/*V*_T_, ventilatory dead space; ${\dot{V}}_{E}$, minute ventilation.

Cardiorespiratory parameters did not differ between hypoxic conditions at rest (TP1: Figure 2 and Table 1; all *P* ≥ 0.199), except for a reduction in *V*_D_/*V*_T_ in HH compared to NH (*P* = 0.028).

GXT data were published previously in full (Vinetti et al., 2024). In brief, peak power output and oxygen uptake (${\dot{V}}_{O_{2}}$) were slightly increased and maximal exercise ${\dot{V}}_{E}$ was moderately increased in HH compared to NH. However, post‐GXT, no difference in cardiorespiratory response, gCBF or $rS_{cO_{2}}$ was evident between the two hypoxic conditions (TP2: Figure 2 and Table 2).

**TABLE 2:** Parameters post graded exercise test in normoxia, normobaric and hypobaric hypoxia; TP2.

	NN [95% CI]	NH [95% CI]	HH [95% CI]	*P* (NH vs. HH)	Effect size
$S_{pO_{2}}$ (%)	94.9 ± 1.3 [94.0, 95.8]	82.8 ± 3.4 [80.5, 85.1]	82.4 ± 3.3 [80.1, 84.7]	0.464	0.12
HR (min^−1^)	93.6 ± 4.8 [90.3, 96.9]	93.4 ± 5.1 [89.9, 96.9]	93.5 ± 5.2 [89.9, 97.1]	0.964	0.01
$P_{\mathrm{ET}O_{2}}$ (mmHg)	115.4 ± 3.3 [113.1, 117.7]	60.8 ± 4.7 [57.5, 64.1]	60.3 ± 3.6 [57.8, 62.7]	0.555	0.13
$P_{\mathrm{ETC}O_{2}}$ (mmHg)	30.0 ± 1.9 [28.7, 31.4]	25.9 ± 1.8 [24.6, 27.2]	25.5 ± 1.3 [24.7, 26.4]	0.271	0.23
RER	0.96 ± 0.10 [0.89, 1.03]	0.83 ± 0.05 [0.79, 0.86]	0.86 ± 0.08 [0.81, 0.91]	0.348	0.47
${\dot{V}}_{E}$ (L min^−1^ btps)	18.0 ± 3.0 [15.9, 20.0]	16.8 ± 3.1 [14.7, 18.9]	16.4 ± 1.5 [15.4, 17.5]	0.764	0.14
*V*_T_ (L btps)	0.87 ± 0.19 [0.74, 1.00]	0.77 ± 0.12 [0.69, 0.86]	0.74 ± 0.11 [0.66, 0.82]	0.371	0.28
*f*_R_ (min^−1^)	21.0 ± 2.7 [19.1, 22.9]	21.9 ± 3.8 [19.3, 24.5]	22.6 ± 3.2 [20.4, 24.8]	0.585	0.18
${\dot{V}}_{E}/{\dot{V}}_{O_{2}}$ (btps)	39.5 ± 3.2 [37.3, 41.7]	40.9 ± 4.5 [37.7, 44.0]	42.1 ± 4.5 [39.0, 45.2]	0.482	0.28
${\dot{V}}_{E}/{\dot{V}}_{CO_{2}}$ (btps)	41.2 ± 2.4 [39.6, 42.8]	49.3 ± 3.0 [47.2, 51.3]	49.1 ± 3.4 [46.8, 51.4]	0.900	0.05
${\dot{V}}_{A}$ (L min^−1^ btps)	12.6 ± 2.4 [10.9, 14.3]	11.4 ± 2.1 [10.0, 12.8]	11.3 ± 0.7 [10.8, 11.8]	0.920	0.05
*V*_D_/*V*_T_ (%)	30.0 ± 4.0 [27.2, 32.7]	31.9 ± 3.2 [29.7, 34.2]	30.8 ± 4.3 [27.9, 33.8]	0.344	0.29
gCBF (mL min^−1^)	737 ± 153 [631, 843]	815 ± 182 [688, 941]	794 ± 139 [698, 891]	0.527	0.13
${\dot{Q}}_{\mathrm{VA}}$ (mL min^−1^)	142 ± 39 [116, 169]	149 ± 47 [116, 182]	141 ± 40 [113, 169]	0.750	0.18
${\dot{Q}}_{\mathrm{ICA}}$ (mL min^−1^)	226 ± 58 [186, 266]	258 ± 72 [220, 298]	256 ± 51 [227, 284]	0.709	0.04
VA TAMV (cm s^−1^)	20.2 ± 2.8 [18.3, 22.2]	19.9 ± 3.4 [17.6, 22.3]	19.7 ± 3.6 [17.2, 22.2]	0.976	0.07
ICA TAMV (cm s^−1^)	25.0 ± 6.0 [20.8, 29.1]	25.1 ± 5.0 [21.7, 28.6]	25.9 ± 5.1 [22.3, 29.4]	0.984	0.15
VA diameter (cm)	0.38 ± 0.04 [0.35, 0.41]	0.39 ± 0.04 [0.36, 0.42]	0.39 ± 0.04 [0.36, 0.41]	0.737	0.18
ICA diameter (cm)	0.44 ± 0.03 [0.42, 0.46]	0.47 ± 0.05 [0.44, 0.49]	0.46 ± 0.03 [0.44, 0.49]	0.907	0.14
$rS_{cO_{2}}$ (%)	—	58.7 ± 4.7 [55.5, 62.0]	57.6 ± 3.5 [55.2, 60.0]	0.385	0.28

Data are presented as means ± standard deviation; *n* = 8 unless otherwise stated. *P*‐values result from comparisons between NH and HH and *P* < 0.05 is considered significant and marked in bold. ES, effect size (Cohen's *d*); CI, confidence intervals (Wald). Abbreviations: *f*_R,_ respiratory frequency; gCBF, global cerebral blood flow; HH, hypobaric hypoxia; HR, heart rate; ICA, internal carotid artery; NH, normobaric hypoxia; NN, normobaric normoxia; $P_{\mathrm{ETC}O_{2}}$, end‐tidal carbon dioxide partial pressure; $P_{\mathrm{ET}O_{2}}$, end‐tidal oxygen partial pressure; ${\dot{Q}}_{\mathrm{ICA}}$, ICA blood flow; ${\dot{Q}}_{\mathrm{VA}}$, VA blood flow; RER, respiratory exchange ratio; $rS_{cO_{2}}$, regional cerebral oxygen saturation; $S_{pO_{2}}$, peripheral oxygen saturation; TAMV, time average mean velocity; TP2, time point 2 (post‐GXT); VA, vertebral artery; ${\dot{V}}_{A}$, alveolar ventilation; ${\dot{V}}_{CO_{2}}$, carbon dioxide output; *V*_D_/*V*_T_, ratio of dead‐space ventilation to tidal volume; ${\dot{V}}_{E}$, minute ventilation; ${\dot{V}}_{O_{2}}$, oxygen uptake; *V*_T_, tidal volume.

In comparison to resting values, gCBF was significantly reduced post‐GXT in HH (TP1 vs. TP2) (944 ± 230 vs. 794 ± 139 mL min^−1^) and NH (883 ± 226 vs. 815 ± 182 mL min^−1^) both *P *< 0.001, respectively (Figure 3), although in HH this gCBF reduction was the result of a reduction in both ${\dot{Q}}_{\mathrm{VA}}$ (TP1 vs. TP2) (157 ± 46 vs. 141 ± 40 mL min^−1^) and ${\dot{Q}}_{\mathrm{ICA}}$ (309 ± 75 vs. 256 ± 51 mL min^−1^) (both *P *< 0.001). This reduction ws led by a marked vasoconstriction in both the VA (TP1 vs. TP2) (0.41 ± 0.03 vs. 0.39 ± 0.04 cm; *P *< 0.001) and the ICA (0.48 ± 0.03 vs. 0.46 ± 0.03 cm; *P* = 0.020), whereas in NH only ${\dot{Q}}_{\mathrm{ICA}}$ was reduced significantly (289 ± 78 vs. 258 ± 72 mL min^−1^, *P* = 0.007), and no change in either extracranial vessel diameter was evident (both *P* ≥ 0.09) (Figure 3). However, pre–post GXT changes in cardiorespiratory variables (including $P_{\mathrm{ETC}O_{2}}$) and cerebral oxygenation ($rS_{cO_{2}}$) were all comparable between hypoxic conditions (*P* ≥ 0.139), except for *V*_D_/*V*_T_, which increased more after GXT in HH than in NH (∆3.1% vs. ∆1.1%, respectively; *P* = 0.039).

**FIGURE 3:**
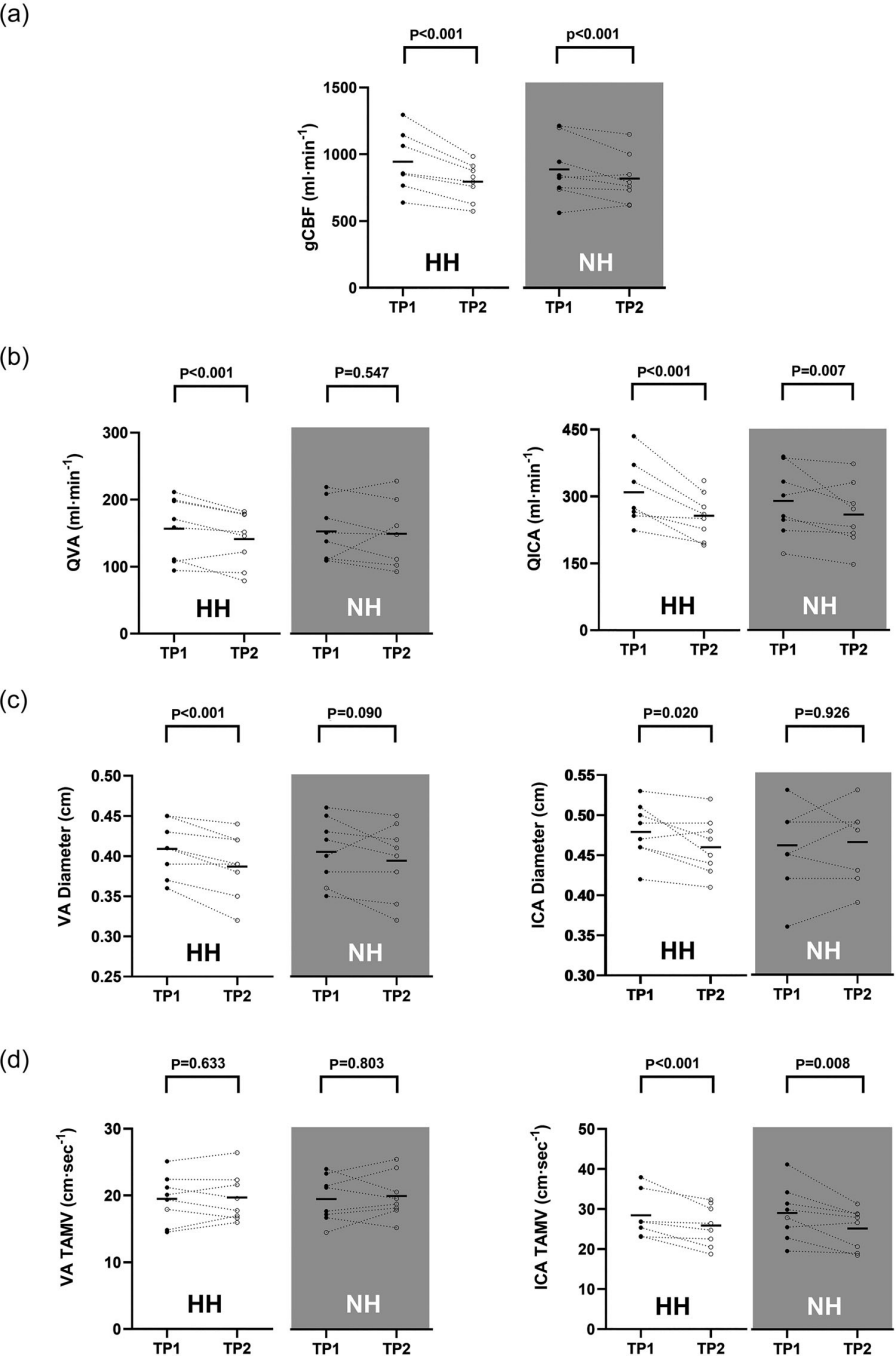
A comparison of cerebrovascular measures before (TP1) and after (TP2) the graded exercise test, in both hypobaric hypoxia (HH) and normobaric hypoxia (NH). (a) Global cerebral blood flow (gCBF). (b) Vertebral artery blood flow (${\dot{Q}}_{\mathrm{VA}}$) and internal carotid artery blood flow (${\dot{Q}}_{\mathrm{ICA}}$). (c) Vertebral artery diameter and internal carotid artery (ICA) diameter. (d) Vertebral artery time average mean velocity (VA TAMV) and internal carotid artery time average mean velocity (ICA TAMV). Small circles represent individual data points, with dotted lines tracking within‐subject changes; *n* = 8 except for HH TP1 ${\dot{Q}}_{\mathrm{ICA}}$, ICA TAMV and gCBF measurements where *n* = 7. Bars represent the mean at each time point. *P*‐values refer to time‐point comparisons (TP1 − TP2) and *P* < 0.05 is considered significant.

## 4 DISCUSSION

The main findings of this study are that, under fully controlled conditions and matched $P_{IO_{2}}$ corresponding to an equivalent altitude of ∼4000 m, at rest, HH elicited a marginally higher gCBF in comparison to NH, despite similar cardio‐respiratory responses. Subtle and contrary to our hypothesis, this augmented vasodilatory response at rest, specifically affecting the anterior circulation, was subsequently abolished post maximal exercise.

### 4.1 HH vs. NH effects on gCBF at rest

Our data demonstrate that, in comparison to NH, in HH gCBF was increased at rest, indicating that cerebrovascular control is affected differentially during acute HH exposure. However, due to negligible changes in concomitant cardiorespiratory parameters, except for a reduced *V*_D_/*V*_T_, the underlying mechanisms remain unclear, and therefore there are interesting points to consider.

One possible explanation for increased gCBF in HH compared to NH is that, due to a reduction in inspired gas density, work of breathing is subtly reduced (Loeppky et al., 1997; Petit et al., 1963), potentiating a comparative reduction in CO_2_ elimination due to a reduced ${\dot{V}}_{E}$ (Conkin & Wessel, 2008; Loeppky et al., 1997; Tucker et al., 1983). In this case, the isolated increase in ICA diameter we observed could have been explained by previous observations of regional heterogeneity in cerebrovascular reactivity to $P_{\mathrm{aC}O_{2}}$ under hypoxic conditions (Ogoh et al., 2013; Sato et al., 2012; Secher, 2013). Given that the total distribution of gCBF to the ICA and VA is ∼72% and 29%, respectively (Zarrinkoob et al., 2015), it is plausible that even a subtle difference in $P_{\mathrm{aC}O_{2}}$ between HH and NH could be further amplified by a larger composite downstream surface area for dilation or indeed differences in cerebrovascular reactivity between conditions (Aebi et al., 2020). However, importantly, aside from the estimated reduction in ventilatory dead space, we found no clear influence of barometric pressure on ${\dot{V}}_{E}$, ${\dot{V}}_{A}$, respiratory pattern or $P_{\mathrm{ETC}O_{2}}$ at rest between HH and NH, which therefore precludes any direct attribution of ventilatory differences to the increase in cerebral vasodilation we observed in HH. Equally, the decrease in ventilatory dead space in HH, in itself a contrasting finding to others (Savourey et al., 2003, 2007), cannot explain the higher gCBF as this argues towards an increase in effective ventilation and therefore a lower $P_{\mathrm{aC}O_{2}}$ in HH compared to NH. The obvious caveats are that without direct measurements of $P_{\mathrm{aC}O_{2}}$, we cannot definitely argue whether physiological dead space was reduced, and by extension our study design precludes our understanding of to what extent this may be a relatively transient finding (Severinghaus & Stupfel, 1957).

Alternatively, previous work has postulated that the physical characteristics of air in HH (i.e., lower gas density) not only potentially alter breathing pattern but also impact pulmonary nitric oxide (NO) turnover. In comparison to acute NH exposure (10 min), expired NO has been reported to be reduced in HH ($P_{IO_{2}}$: 99–75 mmHg) (Hemmingsson & Linnarsson, 2009). The hypothesis is that enhanced axial NO back‐diffusion in HH leads to an associated increase in alveolar uptake of NO into the blood (Kerckx & Van Muylem, 2009; Shin et al., 2004), implying enhanced NO bioavailability (Kayser, 2009). Recent work even goes as far as to indicate that cerebral hypoxic vasodilation is specifically associated with trans‐cerebral haemoglobin‐based *S*‐nitrosothiol (RSNO) release in humans (Hoiland et al., 2023), which could highlight a potential pathway via which bioactive NO could be increased rather than sequestered and thus further reduce cerebrovascular tone in HH and increase cerebrovascular conductance. However, this interpretation directly conflicts with similar work (Faiss et al., 2013), which although confirming a greater and extended (24 h) decrease in expired NO in HH in comparison to NH, concluded there is reduced NO bioavailability due to greater reductions in NO plasma end products (NOx) in NH. Therefore, further work is required to determine whether there is in fact an intrinsic, mechanistic effect of increased NO axial diffusion on NO bioavailability in HH, which could specifically augment cerebrovascular thionitrite release and therefore gCBF. In addition, endothelial mechano‐signaling is redox‐sensitive (Chatterjee & Fisher, 2014), which may render the endothelium more responsive to modest changes in shear stress under hypoxia. This effect could be further accentuated in HH, where previous work suggests a greater oxidative stress burden in comparison to NH (Ribon et al., 2016).

### 4.2 HH vs. NH effects on gCBF post maximal exercise

In response to GXT in NN, the reduction in $P_{\mathrm{aC}O_{2}}$ as the result of relative hyperventilation has previously been shown to result in a decrement in gCBF (Bhambhani et al., 2007; Subudhi et al., 2007). Therefore, we considered it plausible that intense exercise could aggravate potentially subtle differences in resting hypoxia‐induced hyperventilation, further highlighting any difference between NH and HH conditions due to comparatively greater reductions in $P_{\mathrm{aC}O_{2}}$ and therefore gCBF (Imray et al., 2005).

Interestingly, we found that after a GXT the gCBF was the same between HH and NH, even though a higher power output and a greater maximal exercise ${\dot{V}}_{E}$ (due to the lower air density) and greater reduction in $P_{\mathrm{ETC}O_{2}}$ was achieved during the GXT in HH (Vinetti et al., 2024). Similar to the pre‐GXT testing (TP1), post‐GXT (TP2) ventilatory parameters and $S_{pO_{2}}$ were similar between conditions, indicating that subtle differences between the two hypoxic conditions may be overridden by responses elicited by heavy exercise. However, it is also likely that other mechanisms are at play. Indeed, an increased oxidative stress in HH (Faiss et al., 2013; Ribon et al., 2016) would be equally aggravated by the exhaustive exercise component, which could promote the formation of powerful oxidants such as superoxide (O_2_^−^), hydrogen peroxide (H_2_O_2_), hydroxyl radicals (·OH) and even neurotoxic peroxynitrite (ONOO^−^), resulting in increased NO scavenging, greater vascular dysfunction and therefore vasoconstriction (Debevec et al., 2017; Maciejczyk et al., 2022). This could also explain why post‐GXT we observed a marked vasoconstriction in both extracranial arteries (TP1 vs. TP2) in the HH condition only. The cerebrovascular response to exercise under NH and HH remains an important avenue for investigation, which warrants further evaluation to determine the efficacy for an exercise prescription in either NH or HH in individuals pre‐acclimatising for high‐altitude exposures, but also for patients with existing neurovascular disorders.

Overall, the exact mechanisms by which hypobaria may impact cerebrovascular control, especially with the addition of a strenuous exercise component, remain unclear. For a given $P_{IO_{2}}$, factors purported to be differentially affected by hypobaria per se include not only gas density, gas diffusivity and alveolar N_2_ kinetics, but also pulmonary NO turnover, oxidative stress and NO bioavailability. Therefore, overall, these results should be interpreted with caution, but this topic warrants further investigation to determine whether (i) differential hypobaric effects may explain previous reports of augmented prevalence and severity of acute mountain sickness in HH (DiPasquale et al., 2016, 2015; Roach et al., 1996) and (ii) cerebrovascular conductance is altered in HH compared to NH and how this may impact future considerations for therapeutic use cases.

### 4.3 Methodological considerations

Our randomised, single‐blind, crossover study design meticulously matched all environmental conditions, particularly $P_{IO_{2}}$, while avoiding potentially confounding factors (i.e., large equipment dead space) and carefully accounting for potential placebo effects. All subjects were blinded to the condition, including the use of a placebo decompression on entry to the NH condition. Furthermore, in acquiring cerebrovascular data with duplex ultrasound, as opposed to transcranial Doppler, we were able to determine volumetric flow and cerebrovascular conductance as well as changes in flow velocity in both the posterior and the anterior extracranial arteries. All measurement time points were fixed from the start of the simulated ascent, which not only allowed for consistent comparisons between the two hypoxic conditions, but also significant tissue nitrogen wash‐in/washout (Vinetti et al., 2024).

However, there are several limitations to the present study that warrant consideration. The first and most pertinent is the sample size, which was relatively small although comparable to similar studies (Aebi et al., 2020; Loeppky et al., 1997; Roach et al., 1996; Tucker et al., 1983). As a result, although some significant differences were found, the study still might be inadequately powered to detect more subtle, but still scientifically relevant effects. The notable absence of $P_{\mathrm{aC}O_{2}}$ data means we can only speculate on the potential impact of subtle differences in ventilation on gCBF, which are potentially underpowered. Similarly, these findings are limited to an acute bout of hypoxia, which must be considered as the duration of hypoxic exposure is known to impact the results obtained in either condition (Loeppky et al., 1997; Savourey et al., 2003, 2007). Equally, despite an ∼0.5 min higher exercise duration in HH, the time taken from end of GXT to the start of gCBF acquisition was held consistent (8.5 ± 2.1 min). Arguably, due to the non‐steady state physiology post‐GXT, it would have been more appropriate to perform continuous beat‐to‐beat data acquisition of cerebrovascular and cardiorespiratory data and choose an exercise modality that would allow minimal positional changes between the exercise component and gCBF measurements. Equally, the addition of a hypobaric normoxic condition would have been pertinent, especially if oxidative and nitrosative stress were measured. Lastly, it should be acknowledged that the NIRS method used to measure $rS_{cO_{2}}$ captures a composite signal influenced by both cerebral and extracerebral tissues, thereby complicating the interpretation of cerebral oxygenation values in hypoxia, where extracranial perfusion can vary. However, this method, when applied within a crossover design, can still be used to evaluate differences between conditions. However, all are points that warrant future methodological consideration.

### 4.4 Conclusion

We conclude that for a relatively acute exposure, at rest gCBF is increased more in HH for a given $P_{IO_{2}}$, a response that is subsequently abolished post maximal cycling exercise. Although subtle, this differential response again calls into question the interchangeability of these two hypoxic conditions, indicating that cerebrovascular control is differentially affected by NH and HH, despite negligible changes in ventilation. Further, highly controlled comparative studies are required to better understand which are the predominant mechanisms at play and how hypobaria may differentially impact cerebrovascular and neurovascular responses in hypoxia, and potential pathophysiological outcomes at high altitude.

## AUTHOR CONTRIBUTIONS

Hannes Gatterer, Giovanni Vinetti and Rachel Turner conceived and designed the research. Rachel Turner, Hannes Gatterer and Giovanni Vinetti conducted the experiments. Rachel Turner, Hannes Gatterer, Giacomo Strapazzon and Giovanni Vinetti either directly analysed data or contributed to the interpretation of the work. Rachel Turner wrote the first draft of the manuscript. All authors critically read, amended and approved the manuscript. They also all agree to be accountable for all aspects of the work, in ensuring that questions related to the accuracy or integrity of any part of the work are appropriately investigated and resolved if required. All persons designated as authors qualify for authorship, and all those who qualify for authorship are listed.

## CONFLICT OF INTEREST

The authors have no relevant conflicting financial or non‐financial interests to disclose.

## FUNDING INFORMATION

No funding was received for this work.

## Data Availability

The datasets generated and analysed during the current study are available from the corresponding author on reasonable request.
